# Reply to the comment made by Šicho, Vorśilák and Svozil on ‘The Power metric: a new statistically robust enrichment-type metric for virtual screening applications with early recovery capability’

**DOI:** 10.1186/s13321-018-0262-2

**Published:** 2018-03-15

**Authors:** Hans De Winter, Julio Cesar Dias Lopes

**Affiliations:** Laboratory of Medicinal Chemistry, University of Antwerp, Universiteitsplein 1, 2610 Wilrijk, Belgium

The authors of the comment [1] raised an interesting remark about the relation between the power metric (*PM*) [2] and the precision metric (*PR*), also known as the positive predictive value (*PPV*).

In fact, this relation was noted before by the authors of the article that introduced the power metric [2]. Actually, this relationship is shared by all enrichment-type metrics, like the enrichment factor (*EF*) and *ROC* enrichment (*ROCE*), as can be noted by these equations:

1 $$EF = \frac{PPV}{{R_{a} }}$$

2 $$ROCE = \frac{{PPV \cdot R_{i} }}{{\left( {1 - PPV} \right) \cdot R_{a} }}$$

3 $$PM = \frac{{PPV \cdot R_{i} }}{{PPV \cdot R_{i} + \left( {1 - PPV} \right) \cdot R_{a} }}$$

in which *R*_*i*_ and *R*_*a*_ being the proportion of active and inactive instances in the whole dataset with *N* instances:

4 and 5 $$R_{a} = \frac{{n_{a} }}{N}\;{\text{and}}\;R_{i} = \frac{{n_{i} }}{N}$$

with *n*_*a*_ and *n*_*i*_ the number of active and inactive instances in the dataset.

This relationship was one of the reasons to classify the power metric as another enrichment-type metric. In fact, all enrichment-type metrics can be expressed by the same representation:

6 $${\text{`Enrichment-type metric'}} = \frac{TPR}{x}$$

in which the threshold χ will be the cutoff that defines the hitlist of selected compounds. It can be expressed differently for each particular metric:

- in *EF*, χ is the fraction of compounds selected (χ = *N*_*s*_/*N*), related to the number of true and false positives (*TP* and *FP*):
  7 $$\chi = \frac{TP + FP}{N}$$
- in *ROCE*, χ can be related to the fraction of inactive instances wrongly classified as positives:
  8 $$\chi = FPR = \frac{FP}{{n_{i} }}$$
- in *PM*, χ can be related to the sum of the true and false positive rates:
  9 $$\chi = TPR + FPR = \frac{TP}{{n_{a} }} + \frac{FP}{{n_{i} }}$$

Due to these characteristics all these metrics are interconvertible.

A second remark made by Šicho, Vorśilák and Svozil [1] is that the power metric ‘should be accompanied by a metric taking negative classification into account’. We do not entirely agree with this statement as one can estimate all other metrics from the 2-by-2 contingency (confusion) matrix using only the power metric value and the user-defined threshold χ. Combining Eqs. (6) and (9), we can redefine *PM* as a function of χ and *FPR*:

10 $$PM = \frac{TPR}{TPR + FPR}$$

and derive:

11 $$TPR = PM \cdot \chi$$

12 $$FPR = \chi - TPR = \chi - PM \cdot \chi = \chi \cdot \left( {1 - PM} \right)$$

13 $$TNR = 1 - FPR$$

14 $$FNR = 1 = TPR$$

In addition, using the number of actives and inactives, all values of *TP*, *FP*, *TN* (true negatives) and *FN* (false negatives) can be calculated, and from these values any metric can be derived.

The fact that all these metrics are functionally related to the precision metric do not invalidated them as being useful metrics (‘not suitable for performance assessment’, as stated by the authors of the comment). All these metrics have their scopes, strengths and weaknesses. Each one has its meaning and can be used by the user depending on the desired aims. For example, the precision or *EF* metrics might be more appropriate if the user is more concerned about false positives, while in applications with more emphasis on true positive rates the *PM* or *ROCE* metrics would be recommended instead.

In order to have a better understanding on the interpretation of the power metric, lets investigate the dependency of *PM* on threshold χ. In case of a ‘perfect’ screening method in which *FPR* approaches zero, the *PM* tends to approach one (Eq. 10) and *TPR* tends to become equal to χ (Eq. 9). Thus, in this case the maximum value of the *TPR* is limited by the user-defined threshold value χ:

15 $$TPR_{\text{max} } = \chi$$

and the PM could be expressed as:

16 $$PM = \frac{TPR}{{TPR_{\text{max} } }}$$

This leads us to the interpretation of the *PM* as the fraction of active compounds that are correctly predicted in relation to the maximum fraction of active compounds that could be recovered at the chosen threshold χ, or, in other words, *PM* express the probability of an active compound to be correctly classified.
